# Plasmodium falciparum malaria in children at a tertiary teaching hospital: ABO blood group is a risk factor

**DOI:** 10.4314/pamj.v10i0.72205

**Published:** 2011-09-06

**Authors:** John Teye Kuadzi, George Ankra-Badu, Mark Michael Addae

**Affiliations:** 1 Department of Medical Laboratory Sciences, School of Allied Health Sciences, Korle-Bu, Accra, Ghana; 2 Department of Haematology, University of Ghana Medical School, Korle-Bu, Accra, Ghana

**Keywords:** ABO blood groups, malaria, severity, Plasmodium falciparum

## Abstract

**Background:**

ABO blood group antigens are formed by terminal glycosylation of glycoproteins and glycolipid chains present on cell surfaces. Glycosylation modulates all kinds of cell-to-cell interactions and this may be relevant in malaria pathophysiology, in which adhesion has been increasingly implicated in disease severity. This study was done to determine the association between ABO phenotypes and the severity of *P. falciparum* malaria in children.

**Methods:**

One hundred and twenty one children were assessed at the Department of Child Health, KBTH from May to August 2008. ABO blood groups were determined by agglutination. The haemoglobin measurement was done with the haematology analyzer, Sysmex KX-21N. Malaria parasites were enumerated and the presence of malaria pigment noted. Identification of *P. falciparum* was done. Statistical tests used were odds ratio and chi square at a significance level of p<0.05.

**Results:**

24.3% of the 121 children had severe falciparum malaria, and their mean haemoglobin was 4.49 g/dl (SD ±1.69). No significant association was found between the ABO phenotypes and malaria infection (p>0.05). Blood group A was associated with more severe malaria as compared to the blood group O individuals (Odds ratio=0.79, p>0.05); blood group AB (Odds ratio=0.14, p>0.05) and also there was a significant difference in severity of malaria between blood group O and blood group B (Odds ratio=1.28, p>0.05).

**Conclusion:**

Non-O blood group children are more prone to severe malaria caused by *P. falciparum* malaria than the group O, despite the lack of significant association between ABO blood groups and *falciparum* malaria.

## Background

ABO blood groups are carbohydrate histo-blood antigens that are also expressed in many tissues and have important roles in modulating protein activities both in infection and in some types of cancer [1]. These antigens are formed by terminal glycosylation of glycoproteins and glycolipid chains present on cell surfaces. Glycosylation modulates all kinds of cell-to-cell interactions and this may be relevant in malaria pathophysiology, in which adhesion has been increasingly implicated in disease severity [2]. Blood group A has been reported as a risk factor for severe malaria [3], and as a co-receptor for *P. falciparum* resetting [4], whereas blood group O may offer some protection against severity of disease [5]. There is increasing evidence that both the risk of acquiring P. falciparum infection, and the risk of developing severe complications are determined by host genetic factors [6]. The protective role of several erythrocytic variants, some of them related to blood groups, is one of the best examples of this genetic modulation [7]. The others include haemoglobins S, C and E, α and β thalassaemias, Glucose-6-phosphate dehydrogenase deficiency, Southern Asian Ovalocytosis, and Glycophorins A, B and C variants, all of which influence malaria pathogenesis [8].

Malaria is the most important parasitic infection of man, and is associated with a huge burden of morbidity and mortality in many parts of the tropical world. Mortality rates of 10-30% have been reported among children referred to hospitals with severe malaria, although these rates are even higher in rural and remote areas where diagnosis and treatment are not readily available [9]. Over 40% of the world's children live in malaria-endemic countries. Each year, approximately 300 to 500 million malaria infections lead to over one million deaths, of which over 75% occur in African children under 5 years infected with *P. falciparum* [10]. Malaria accounts for one in five of all childhood deaths in Africa. Frequent consequences of malaria anaemia, low birth-weight, epilepsy, and neurological problems compromise the health and development of millions of children throughout the tropical world [11]. Severe and complicated malaria is usually caused by delay in treating an uncomplicated attack of *P. falciparum* [12].

### The immune system: parasite survival strategy and disease

It is helpful to consider the multifaceted nature of the interaction between the host immune system and the parasite. Central to this interaction are cytokines that are released by immunocompetent cells in a highly regulated fashion [13]. They participate in the control of all immunologically relevant events, whether they concern either activation, proliferation, and subsequent effector functions of recirculating immunocompetent cells or regulation of cells residing in tissues (for example, resident mononuclear phagocytes and endothelial cells). It has been established that cytokines not only participate in the qualitative (for example, antibody isotype switch) and quantitative regulation of the immune response but also participate in many other complex processes such as hematopoiesis and pregnancy. During the erythrocytic cycle, soluble products of *Plasmodium* spp. known as malarial toxins direct systemic release of proinflammatory cytokines (for example, tumor necrosis factor-o^∼^ (TNF-ix)) which act on many other cellular systems such as endothelium. Equally important are parasite antigens, which stimulate T cells to directly secrete or induce production of cytokines from other cells. Before *P. falciparum* infection, many individuals have *P. falciparum* reactive T cells, often at high frequency. Such parasite-reactive T cells have probably arisen as a result of antigenic cross reactivity between environmental organisms and parasite-derived molecules [14].

Incubation of blood mononuclear cells with parasitized erythrocytes can drive proliferation of these T cells even when the parasitaemia is as low as one parasite per microliter of blood. Because many of these T cells secrete interferon y (IFN-y) and other cytokines and can facilitate production of TNF-o^∼^ by monocytes, they have the potential to be involved in disease pathogenesis. Both IFN-^∼^ and TNF-cl may play roles in dyserythropoietic anemia, and TNF-a may contribute to cerebral malaria as a result of up-regulation of intercellular adhesion molecule-1 (ICAM-1) in cerebral blood vessel endothelium. With respect to TNF-et production, T cells--whether they express y8 [15] or oil8 T cell receptors may play as important a role in disease pathogenesis as that postulated for the direct stimulation of mononuclear phagocytes by malarial toxins. It is likely that parasite-dependent activation of T cells and mononuclear phagocytes leads not only to disease but also to killing of parasites. Whether all of the cross-reactive T cells, once activated during the primary infection, will remain functional during a persistent infection or until exposure to re-infection deserves to be studied. The parasite also has other strategies for interacting with the immune system, including the following: antigenic variation; (it) a still undefined, splenic dependent regulation of parasite genes encoding structural proteins and adhesive molecules on the erythrocyte surface that are involved in adherence to endothelium; and low immunogenicity, of conserved parasite peptides that are targets of antibodies able to interfere with parasite survival.

### Possible mechanisms to explain *P. falciparum* selective genetic pressure in favour of group O and against group A

The adherence of parasitized RBCs to other cells is central to the pathophysiology of severe malaria syndromes including cerebral malaria, respiratory failure, multi-organ failure and death [16,17]. Parasitized RBCs adhere to the vasculature through a process termed “sequestration” closely mimicking inflammatory leukocyte attachment [18]. Furthermore, half of infected RBC isolates51 form occlusive intravascular aggregates, which consist not only of infected RBCs bound to each other (“autoagglutinates”) [19], but also of infected RBCs bound to uninfected RBCs (“homotypic RBC rosettes”) [20] and/or to platelets (“heterotypic RBC rosettes”) [21]. Sequestration and rosette formation impair blood flow, causing tissue ischemia and cell death [22]. Not surprisingly, in-vitro rosetting is more pronounced in parasite strains derived from patients with severe disease, particularly in cases of cerebral malaria [23–26]. *P. falciparum* is unique among malaria species in that infected erythrocytes express an adhesive determinant, termed *Plasmodium falciparum* Erythrocyte Membrane Protein-1 (PfEMP-1). PfEMP-1 is encoded by the parasite genome and is expressed on the outer surface of infected RBCs. PfEMP-1 binds to a variety of target molecules found on RBCs, platelets, and vascular endothelial cells [16,17]. The structure of PfEMP-1 includes variable numbers of two types of adhesive binding domains: Duffy binding-like (DBL) regions and cysteine-rich interdomain regions (CIDR). DBL-1α demonstrates lectin-like properties, causing it to bind primarily to cells bearing A and B blood group oligosaccharides, and to other glycosylated targets, such as the glycoprotein CD35 (CR1), and heparan sulfate-like glycosaminoglycan [27,28]. CIDR1, on the other hand, binds principally to CD36 (Platelet Glycoprotein IV), thus targeting platelets and endothelium.

It has been established that parasitized erythrocytes form rosettes more readily with red blood cells of A, B, or AB groups than with cells of blood group O. This parasite-triggered RBC rosette formation is associated with severity of malaria disease [14]. Rosette formation and autoagglutination are adhesive phenotypes associated with disease severity in *P. falciparum* malaria [29]. The adherence of parasitized RBCs to other cells is central to the pathophysiology of severe malaria syndromes including cerebral malaria, respiratory failure, multi-organ failure and death [16,17].

There is increasing evidence that *P. falciparum* malaria is influenced by ABO blood group but the extent of association between both is yet to be well defined. *P. falciparum*, the most dangerous or deadly of the four human malaria parasites, causes about 90% of all malaria deaths in the world today especially in sub-Saharan Africa [9,20]. However, no risk associated data for ABO phenotypes and *P. falciparum* malaria has been reported to date, in Ghana. Thus, this study investigated the hypothesis that ABO blood groups are associated with the severity of *P. falciparum* infection and the results showed that non-O blood group children are more prone to severe malaria caused by *P. falciparum* than the group O children.

## Methods

### Ethics

This study was approved by the Ethics Review Committee of the School of Allied Health Sciences, College of Health Sciences; Korle-Bu. Parental/guardian consent for child participation was sought during the study.

### Study Site and Sample Size

The study was carried out at the Department of Child Health, Korle-Bu Teaching Hospital (KBTH) from May to August 2008. The KBTH is the largest tertiary hospital in Ghana with 17 departments and 1500 beds. One hundred and twenty one (121) children between 3 months and twelve years were sampled.

### Inclusion criteria

Patient between 3 months and twelve years old, whose blood film (BF) test was reported as malaria parasites present and thin film showed *Plasmodium* specie to be *falciparum*.

### Exclusion criteria

Patient below 3 months old or twelve years and above; whose blood film (BF) test was reported either as no malaria parasite present or malaria parasites present and thin film showed *Plasmodium* specie to be either *falciparum* or *malariae*.

### Blood collection and staining

Two ml venous blood from the patient was collected into an EDTA-anticoagulated tube (1.2mg of anhydrous dipotassium salt) using a 2ml syringe and needle set. Two drops of blood were placed on a clean glass slide and a thick film and a thin film were prepared on the same slide and allowed to air dry. The thin film was fixed with methanol for one minute. The slides were placed in a staining rack and immersed in a staining trough. Giemsa stain (Wako, Japan) diluted 1 in 10 with phosphate buffer pH 7.2 was used to stain the film for 10 minutes. The stain was washed off the slides using the buffer, pH 7.2. The back of the slides were wiped dry and placed in a draining rack for the film to air-dry.

### Microscopy and parasite density determination

The parasite density was counted as parasite numbers per microlitre of blood by counting parasites against white blood cells. Using the x100 objective, 200 WBCs were counted systematically, estimating at the same time the numbers of parasites (asexual) in each field covered, with two tally counters.

Calculation = WBC count/µl of patient X Parasites counted against 200 WBCs/200

The above formula was used if after 200 WBCs have been counted, and 10 or more parasites were identified. However, if after 200 WBCs have been counted, and 9 or fewer parasites have been counted, counting continued until 500 WBCs have been counted and the number of parasites was recorded.

### Severity of disease

Severity of the *Plasmodium falciparum* malaria disease was determined based on the parasite density calculated as above. **Severe parasitaemia:** was defined as hyperparasitaemia (> 250,000 parasites/µl or more than 10% of red cells are parasitized); and/or severe anaemia (Haemoglobin concentration of<5g/dl). **Moderate parasitaemia:** was defined as parasitaemia between 51,000-249,000 parasites/µl and/or Haemoglobin concentration between 5-8g/dl. **Mild parasitaemia:** was defined as parasitaemia of 1,000-50,000 parasites/µl and/or Haemoglobin concentration>8g/dl.

### ABO grouping

The spun tube technique [30], a liquid-phase system, was employed for the detection of ABO antigens and antibodies using cell and serum grouping. Four small tubes (75×12 mm) were labeled 1 to 4. Each tube was filled as follows:

Tube 1-1 volume anti-A serum and 1 volume 5% patient's red cell suspension in saline
Tube 2-1 volume anti-B serum and 1 volume 5% patient's red cell suspension in saline
Tube 3-1 volume patient's serum and 1 volume 5% A cells in saline
Tube 4-1 volume patient's serum and 1 volume 5% B cells in saline

The contents of the tubes were mixed by gently tapping the base of each tube with the finger. The tubes were left at room temperature for 5 minutes and then centrifuged at 150g for a minute. The results were read by gently tapping the base of each tube, looking for agglutination or haemolysis. Red cell reagents (known A, B and O cells) were used to control the antisera (A and B). An autocontrol was also set i.e. patients red cells were added to their own serum to detect the possible presence of autoagglutination. The haemoglobin measurement and leucocyte count were done with an automated haematology analyzer, Sysmex KX-21N. The Sysmex KX-21N employs DC detection method for the leucocyte count and Non-cyanide Sodium Lauryl Sulfate (SLS) method for the haemoglobin measurement.

## Results

The basic demographic data of the 121 children is presented in Table 1. ABO phenotypes distribution was observed as A – 24 (19.8%), B – 32 (26.4%), AB – 5 (4.2%) and O – 60 (49.6%) among the 121 children. Twenty-six (24.3%), of the one hundred and twenty one (121) children assessed were diagnosed to have severe falciparum malaria (Table 2), who had a mean age of 2.2 years (SD ±1.8), mean leucocyte count of 11.5±4.5 and mean haemoglobin concentration of 4.5 g/dl (SD ±1.7) (Table 3).

**Table 1:** Age distribution, haemoglobin concentration, total white cell count (WBC) and ABO blood groups distribution among the subjects in the study at the Department of Child Health, Korle-Bu Teaching Hospital, Ghana

					Blood groups			
Gender	**Number**	**Mean Age (years)**	**Mean Hb (g/dl)**	**Mean WBC (x 10^9^/l)**	**A**	**B**	**AB**	**O**
**Male**	71 (59%)	3.6±2.8	7.9±2.6	10.8±5.6	14	18	4	35
**Female**	50 (41%)	3.3±2.8	7.3±2.6	11.5±4.8	10	14	1	25

**Table 2:** ABO blood groups and distribution of *P. falciparum* malaria based on severity of parasitemia among the subjects in the study at the Department of Child Health, Korle-Bu Teaching Hospital, Ghana

Parasitemia				

Phenotypes	*Severe	^+^Moderate	^#^Mild	Total
A	6 (23%)	7 (18%)	11 (20%)	24
B	5 (19%)	12 (30%)	15 (27%)	32
AB	3 (12%)	1 (2%)	1 (2%)	5
O	12 (46%)	20 (50%)	28 (51%)	60
Total	**26**	**40**	**55**	**121**

* Severe parasitaemia was defined as hyperparasitaemia (> 250,000 parasites/µl or more than 10% of red cells are parasitized); and/or severe anaemia (Haemoglobin concentration of<5g/dl).

^+^ Moderate parasitemia was defined as parasitaemia between 51,000 – 249,000 parasites/µl and/or Haemoglobin concentration between 5-8g/dl.

^#^ Mild parasitemia was defined as parasitaemia of 1,000 – 50,000 parasites/µl and/or Haemoglobin concentration>8g/dl

**Table 3:** Mean total white cell count (WBC) and haemaglobin concentration distribution among the children in relation to the severity of malaria parasitemia, Korle-Bu Teaching Hospital, Ghana

Severity of malaria parasitemia	WBC±SD (x10^9^/l)	HB±SD (g/dl)
**Mild**	11.5±6.1	9.9±1.3
**Moderate**	11.4±6.2	6.4±0.8
**Severe**	11.5±4.5	4.5±1.7

There was no significant association between the ABO phenotypes and parasitemia (χ^2^=5.47, p>0.05). Blood group A was associated with severe malaria when it was compared with individuals with blood group O (Odds ratio=0.79, p>0.05, 95% CI: 17,593-96,306) (Table 4). Moreover, blood group AB was associated with more severe falciparum malaria when compared to blood group O (Odds ratio=0.14, p>0.05, 95% CI: 18,582-119,498) and also there was a significant difference in severity of malaria between blood group O and blood group B (Odds ratio=1.28, p>0.05, 95% CI: 11,332-82,716) (Table 4).

**Table 4:** The determination of how significant the difference between the O blood group and the non-O blood groups (A,B and AB) in the distribution of the severity of the *P. falciparum* malaria in the children at the Department of Child Health, Korle-Bu Teaching Hospital, Ghana

Parasitemia					

	Severe & Moderate Parasitemia	Mild Parasitemia	Odds ratio (O versus Non-O)	P-value	Difference in Severity
A	13	11	0. 97, 95% C.I. (17,593 – 96,306)	0.5	Not Significant
B	17	15	1.01, 95% C.I. (11,332 – 82,716)	0.7	Not Significant
AB	4	1	0.29, 95% C.I. (18,582 – 119,498)	0.5	Not Significant
O	32	28	N/A	N/A	N/A

## Discussion

The ABO blood group system in Ghanaians and other West Africans from earlier studies have given the prevalence of blood group O as 50%, AB about 3%, and the remainder being groups A and B in about equal proportions with B being slightly more than A [31–34]. The same observation was made in this study except that the AB group was higher than reported frequencies.

Studies conducted show that parasitized erythrocytes form rosettes more readily with red blood cells of either A, B or AB groups than with those belonging to blood group O,[3,35,37] and that, in Zimbabwe, blood group A was associated with both lower hemoglobin levels and severe central nervous system malaria with coma [4]. In spite of the fact that these phenomena might provoke an association of ABO blood groups and severe falciparum malaria infection, the results presented in this study do not permit the rejection of the null hypothesis that the ABO blood group system is not associated with *P. falciparum* malaria infection.

The mechanism by which blood group O confers the somewhat protective effect against severe malaria compared to blood groups A, B, or AB is not totally understood as indicated by studies reviewed [38]. However, a logical explanation may be provided on the basis of the rosette formation. Evidence from some studies reviewed clearly established that parasitized erythrocytes form rosettes more readily with RBCs of either A, B, or AB blood groups than with those belonging to blood group O [3,35–37]. Also, it is well established that this parasite-triggered RBC rosette formation is associated with the development of cerebral malaria [20,25].

In this study, there was no significant association between the ABO blood group system and *P. falciparum* malaria infection, as observed in Nigeria among children with severe malaria [39, 40]. It was observed that the non-O blood group individuals tend to have more severe falciparum malaria than the blood group O individuals. Moreover, in the severe malaria group, it was noted that the lowest mean haemoglobin occurred in the blood group A individuals, as observed in a trial in Zimbabwe [4]. The two cases of very high risk of mortality (more than 10% of red blood cells parasitized or more than 500,000 parasites/µl) were observed in individuals with blood group O whereas the percentage severity of falciparum malaria was highest with the AB phenotypes. And this seems to be significant because the percentage (11.5%) was higher than the frequency distribution (4.2%) among the children assessed.

## Conclusion

This study has shown that non-O blood group children are more prone to severe malaria caused by *P. falciparum* than the group O children. Therefore, identifying ABO phenotypes could help to detect those children at high risk for malaria.
